# The impact of family support on depression among primary and secondary school teachers in China: the serial mediating roles of subjective time pressure and work-family conflict

**DOI:** 10.1186/s40359-025-03543-w

**Published:** 2025-11-19

**Authors:** Shuang Zhong, Jinyu Hu, Huachun Xu

**Affiliations:** 1 Institute of Education and Science, Leshan Normal University, Leshan, China; 2 Sichuan Teacher Development Center, Chengdu, China; 3 The Department of Psychology, Sichuan Normal University, Chengdu, China

**Keywords:** Family support, Depression, Subjective time pressure, Work-family conflict, Teachers

## Abstract

**Background:**

The mental health of teachers has been demonstrated to be a contributing factor to the promotion of high-quality education in the country. The present study draws upon Conservation of Resources Theory and Social Support Theory in order to explore the relationship between family support and teachers’ depression in primary and secondary school teachers, along with its underlying mechanisms.

**Methods:**

This study employed convenience sampling to survey 3,159 primary and secondary school teachers (*M*_age_ = 40.43, *SD* = 10.22; 37.4% male) in China via online platforms. Participants completed the Family Support Questionnaire, Time Affluent scale, Teacher Work-Family Conflict Questionnaire, and Patient Health Questionnaire. The subsequent analysis of the data was conducted using SPSS and its PROCESS plugin.

**Results:**

The results suggested that family support was significantly negatively associated with teachers’ depression. Furthermore, subjective time pressure and work-family conflict exerted a serial mediating effect in the relationship between family support and depression in this teacher group.

**Conclusions:**

The findings of the study indicate that teachers who encounter diminished levels of familial support are more prone to manifest elevated degrees of depression. This phenomenon may be attributed to the fact that such teachers face greater subjective time pressure and more intense work-family conflicts, which exacerbate depression. Therefore, the provision of enhanced family support and encouragement has been demonstrated to be an effective means of alleviating the stress and depression experienced by these teachers, thereby promoting their mental health.

## Introduction

Depression is a global health concern characterised by persistent low mood, anhedonia, and feelings of low self-worth [3]. A national survey indicates that the prevalence of moderate to severe depression in China stands at 8.29% [28], translating to over 100 million people in the country living with the condition. Within this context, depression among teachers is particularly prominent. Teachers have been found to be at an elevated risk of developing common mental disorders in comparison to individuals engaged in other occupations [26]. A substantial body of research has revealed that the prevalence of psychological problems and depression among the population of primary and secondary school educators in China is higher than that observed among other groups. Furthermore, this prevalence has been observed to exhibit a gradually increasing trend [11, 24, 48]. Depression has been shown to have a detrimental effect on teachers' physical and mental health, increasing their risk of all-cause mortality [1, 44]. In addition, it has been demonstrated that depression can also have a negative impact on students' academic performance and overall well-being [9, 46].

The phenomenon of teacher depression is understood to emerge from a complex interplay of individual, social, and organisational factors [16]. In research, family factors are frequently incorporated into the category of social factors, potentially resulting in an underestimation of their influence. Recent studies have revealed that family support and social support exert distinct effects on individual health, with family support having a more significant impact on mental health than social support [4]. It is important to note that the prevailing practice of conceptualising family factors as a subset of social factors may potentially diminish their distinct impact. Thus the exploration of the factors that influence depression in the primary and secondary teaching profession, as well as the underlying mechanisms that underpin it, is of significant theoretical value and practical significance for enhancing teachers' mental health, advancing educational development, and building a strong educational nation.

Whilst there has been a considerable body of research exploring the factors that influence teachers' mental health, including work-related variables (e.g., workload intensity, school leadership support) and individual factors (e.g., emotional regulation, personality traits), there has been comparatively little focus on the role of family systems in this process [12]. Despite the recognition among scholars of the significance of family support in the context of teachers' mental health, this aspect is predominantly addressed within the broader framework of social support in extant literature. The examination of family support as a standalone entity, with a specific subject on its impact on psychological well-being, remains limited. The underlying mechanisms that facilitate this impact are not yet fully comprehended. Given that family and work represent two of the most significant domains in an individual's life, the relationship between them, including the mediating mechanisms that connect family support to mental health outcomes, warrants further scholarly exploration.

### Family support and teachers’ depression

Social support theory posits that diverse forms of support (e.g., emotional, informational, and instrumental) can effectively mitigate the adverse impacts of stressful events, thereby enhancing individuals' quality of life and mental well-being [5]. In China, deeply rooted collective values endow the family with a distinctive influence on individuals' mental health. A substantial body of research has highlighted the pivotal role of family support in shaping an individual's quality of life and well-being [4]. In the context of teachers' work, family support can be defined as various forms of assistance and backing provided by the family sphere that are conducive to the lives of teachers [31]. Sun et al. [41] investigated the relationship between family support and depression, concluding that lower levels of family support were associated with higher rates of depression in adulthood. Moreover, extant research has identified a significant negative correlation between family support and depression among both adolescent and older age groups [30, 45]. The present study proposes the following hypothesis: a significant negative correlation exists between family support and depression among primary and secondary school teachers.

### Mediating roles of subjective time pressure

Subjective time pressure is defined as the subjective experience in which individuals feel they lack sufficient time to accomplish what they intend to do [27, 38]. The Conservation of Resources (COR) theory posits that individuals, with inherently limited resources, are driven to acquire, retain, protect, and cultivate them. Stress emerges when resources are threatened, lost, or when investments in them fail to yield anticipated returns. For example, stress may result from a continuous depletion of personal resources, such as time and energy, without adequate replenishment. This stress has been shown to impair physical and mental health sequentially [18]. Research has demonstrated that educators often work beyond their standard working hours [11, 34]. Consequently, when teachers' time resources are continuously depleted and they receive insufficient family support, they may experience more intense subjective time pressure. Cohen and Wills' [6] study observed that social support can mitigate individuals' perceived stress levels, suggesting that family support may effectively alleviate teachers' subjective experience of time pressure. Conversely, teachers in primary and secondary school may encounter intensified subjective time pressure amid inadequate family support.

However, the COR theory posits that elevated subjective time pressure may result in diminished subjective well-being and mental health [25]. Roxburgh's [37] study demonstrated that heightened subjective time pressure can elevate individuals' depression levels, amplify negative emotions, and thereby impair mental health. Therefore, the role of subjective time pressure as a mediator in the relationship between family support and teachers' depression is also a core of this study.

### Mediating roles of work-family conflict

Work-family conflict (WFC) is defined as a form of incongruence between work and family role pressures, wherein the demands of one role hinder involvement in the other [14]. The domains of work and family are widely regarded as the two most critical aspects of an individual's life. So the role of work-family conflict in the relationship between family support and teachers' depression merits investigation. The Job Demands-Resources model (JD-R) posits that individuals possess job resources, comprising internal resources (e.g., cognitive traits, behavioral patterns) and external resources (e.g., organizational support, family support, co-worker support). In instances where external resources prove inadequate, individuals may encounter challenges in coping with environmental demands, leading to unsuccessful goal attainment and subsequent repercussions on their physical and mental well-being [10]. Research has demonstrated that social support is a significant negative predictor of individuals' work-family conflict [17, 35], such that greater social support received by an individual corresponds to lower levels of work-family conflict.

Work and family represent two of the most vital domains in an individual's life. Conflict between them can exert substantial negative effects on teachers' physical and mental health. A substantial body of research has consistently demonstrated that heightened family-work conflict among teachers is significantly associated with an increased likelihood of psychological issues, including burnout, depression, and anxiety [36, 42, 46]. In summary, the third hypothesis of this study is proposed as follows: teachers' perceived family support impacts their depression levels through its effect on work-family conflict.

### Subjective time pressure and work-family conflict

The relationship between subjective time pressure and work-family conflict can be interpreted through the COR and role theory. Time is a valuable and finite resource. Given that teachers generally work longer hours than the average employee, a reduction in objective time is likely to intensify their subjective time pressure. According to role theory, individuals assume multiple roles, such as teacher, parent, and child, each of which is associated with distinct expectations. Divergent role demands have been shown to lead to role conflict, which has been demonstrated to exert a range of negative effects on well-being [23, 32]. Work-family conflict has been identified as a key mechanism in this process. As a result, elevated subjective time pressure among teachers may intensify work-family conflict, thereby contributing to a vicious cycle of pressure, conflict, and depression. Given that teachers' subjective time pressure primarily originates from the domains of work and family, an escalation in such pressure is likely to amplify their experience of work-family conflict.

### The present study

The theory of COR posits that the resources possessed by individuals exist in the form of interconnected resource reservoirs, where various resources are interrelated. These resources are embedded within diverse contexts that have the capacity to either create or deplete them. The available resources for individuals are largely shaped by external environmental factors, including family, community, and culture [19–21]. The JD-R model was developed as a more work-specific theoretical framework, drawing on the COR theory. The JD-R postulates that work environments are comprised of two key elements: job demands, which deplete personal resources, and job resources, which provide support and foster motivation [2, 10]. For this reason, the COR and JD-R theories provide a robust theoretical framework for the present study's examination of the relationships among family support, subjective time pressure, work-family conflict, and teacher depression. The present study investigates the relationship between family support and teachers' depression in China, while exploring the roles of subjective time pressure and work-family conflict within this framework. The research hypotheses are outlined as follows.

- Hypothesis 1 Family support were negatively associated with teachers’ depression in primary and secondary school.
- Hypothesis 2 Subjective time pressure mediates the relation between family support and depression.
- Hypothesis 3 Work-family conflict mediates the association between family support and depression.
- Hypothesis 4 Subjective time pressure is positively associated with teachers’ work-family conflict.

### Methods and procedure

The present study was conducted in accordance with the principles delineated in the Declaration of Helsinki, which governs experiments involving human subjects. On the 12th of June 2024, the Ethics Committee of Leshan Normal University conducted a comprehensive review (ID: LNU-20240612a). The present study obtained electronic informed consent from primary and secondary school teachers. This study employs an online survey approach, whereby questionnaires were disseminated to educators in primary and secondary school throughout the province via the Sichuan Teacher Development Center. Prior to participating in the survey, all subjects were provided with comprehensive information concerning the study's purpose, the voluntary basis of their participation, their right to withdraw at any point, and assurances regarding data anonymity and confidentiality. The participants received neither incentives nor rewards.

A total of 3,359 teachers participated in this study. Following the exclusion of questionnaires that were deemed invalid due to response times that were excessively brief, repeated submissions, and other such issues [49], a total of 3,159 valid questionnaires were retained, yielding an effective response rate of 94.05%.

### Participates

Of the 3,159 participants (*M*_age_ = 40.43, *SD* = 10.22), 1,183 were male and 1,976 were female. The marriage distribution was as follows: married (*n* = 2489), single (*n* = 507), other marital statuses (*n* = 163). The survey collected information on whether teachers work in middle schools or primary schools. There were 1,439 primary school teachers and 1,720 secondary school teachers. Regarding the school location, 1,734 participants work in rural schools, and 1,425 work in urban schools. There were There were 21 people with educational qualifications at or below senior high school level, 535 people with college degrees, 2542 with bachelor’s degrees, and 61 with master's degrees.

### Measurements

#### Family support

The Chinese version of the Work-Family Support scale was developed by Li and Zhao [31]. The scale has been validated in the Chinese context [47]. The scale is single-dimensional and consists of 10 items, each rated on a scale from 1 (strongly disagree) to 5 (strongly agree), where higher scores indicate greater family support. In this study, the overall consistent Cronbach's alpha is 0.933.

#### Subjective time pressure

The Time Affluent scale, developed by Kasser and Sheldon [25], was utilized to assess subjective time pressure. This version of the Time Affluent scale has been validated in a sample of Chinese adults [51]. The scale under consideration comprises eight items, each of which is measured on a Likert-type scale ranging from 1 (strongly disagree) to 5 (strongly agree). Items 2, 4, 5, and 7 are reverse-scored, with higher scores indicating greater subjective time pressure. The scale demonstrated substantial internal consistency in this study, with Cronbach's alpha reaching 0.819.

#### Work-Family Conflict

The Work-Family Conflict measure for primary and secondary school teachers in China was developed by Wu et al. [46]. This scale underwent a process of cultural adaptation for Chinese teachers in primary and secondary school, and its reliability and validity have been well established in prior studies [8]. The questionnaire under consideration comprises 22 items, which are distributed across two dimensions. The Work Interference with Family (WIF) and Family Interference with Work (FIW) scales each consist of 11 items, which are used to measure work interference with family and family interference with work, respectively. Participants were asked to respond to the extent of their agreement or disagreement with a series of statements using a 5-point Likert scale ranging from 1 (strongly disagree) to 5 (strongly agree). Higher scores on this scale indicate stronger work-family conflict. In the present study, the overall internal consistency (Cronbach's alpha) was 0.962, with Cronbach's alpha of 0.939 for WIF and 0.964 for FIW.

#### Depression

The Chinese version of the Patient Health Questionnaire-9 (PHQ-9) was originally developed by Spitzer et al. [40] and revised by Kroenke et al. [29]. The Chinese version of PHQ-9 has been validated in a sample of Chinese teachers (Peng et al., 2025). The questionnaire under consideration contains 9 items and utilizes a 4-point scoring scale, where 0 indicates "not at all" and 3 indicates "nearly every day”. Higher scores on the PHQ-9 are indicative of more severe depression. In the present study, the internal consistency reliability of the scale was 0.926.

#### Data analysis

To ensure the quality of the responses, a series of precautions were implemented prior to the distribution of the questionnaire. These precautions included the collection of anonymous data, the incorporation of lie-detection items, and the implementation of reverse scoring for a selection of items. Harman’s single-factor test revealed that the largest eigenvalue accounted for 35.37% of the variance in the current study, a value below the 40% threshold [43, 50]. The model fit indices in AMOS consistently indicated a poor fit for the single-factor model (χ/*df* = 41.08, RMSEA = 0.138, CFI = 0.0.437, TLI = 0.412, SRMR = 0.156). This finding suggests that the present study is not subject to common method bias. Therefore, the data are deemed suitable for subsequent analyses.

The data analysis in this study followed three sequential steps: (1) Descriptive statistical analyses and group comparisons were performed using SPSS 22.0. Specifically, independent samples t-tests and one-way ANOVAs were employed to examine differences in key variables across demographic characteristics. (2) Partial correlation analyses were conducted to explore the associations between family support, subjective time pressure, work-family conflict, and depression among primary and secondary school teachers, with demographic variables controlled for as covariates. (3) The mediation analyses were executed using the PROCESS macro (Version 3.5; [15]) with a bootstrap method to evaluate the study hypotheses.

## Results

### Descriptive statistics and partial correlation analysis

Table 1 represents the mean scores of participants and the partial correlations between the analyzed variables. A correlation matrix revealed significant intercorrelations among all key variables, thereby providing a preliminary foundation for the evaluation of the research hypotheses.

**Table 1:** Descriptive statistics and partial correlation analysis of variables

	*M* ± *SD*	1	2	3	4
1 Family Support	3.52 ± 0.95	1			
2 Subjective Time Pressure	3.46 ± 0.74	−0.343^***^	1		
3 Work-Family Conflict	2.07 ± 0.97	−0.276^***^	0.206^***^	1	
4 Depression	0.91 ± 0.67	−0.376^***^	0.385^***^	0.516^***^	1

^***^p < 0.001

### The serial mediating model

To ensure the robustness of the results, all variables were mean-centered prior to conducting the mediation analysis. For the serial mediation analysis, Model 6 of the SPSS PROCESS macro (Version 3.5; [15]) was employed. The following covariates were included in the analysis: teachers' age, gender, household registration status, marital status, parental status, whether they held a class teacher position, and annual household income. These variables exhibited significant group differences.

The findings of the study indicated a negative and significant correlation between family support and teachers' depression levels (β = ﹣0.375, *t* = ﹣22.797, *p* < 0.001). Subsequent mediation analysis revealed that teachers' subjective time pressure and work-family conflict partially mediated the relationship between family support and depression. The detailed results are presented in Table 2.

**Table 2:** Standardized regression coefficients among research variables

Variables	Subjective time pressure			Work-family conflict			Depression		
	*β*	*t*	95% CI	*β*	*t*	95% CI	*β*	*t*	95% CI
family support	﹣0.34	20.48^***^	[−0.37, −0.31]	﹣0.23	﹣13.18^***^	[−0.259, −0.192]	﹣0.17	−11.28^***^	[−0.20, −0.14]
Subjective time pressure				0.30	17.45^***^	[0.268,0.336	0.15	9.75^***^	[0.121,0.182]
Work-family conflict							0.47	30.50^***^	[0.437,0.497]
*R*^2^	0.16			0.21			0.41		
*F*	72.67^***^			90.64^***^			220.35^***^		

For simplicity, some covariables (i.e., gender, age, and marriage status) are not displayed

The mediation analysis results revealed that subjective time pressure and work-family conflict exerted a serial mediating effect on the relationship between family support and teachers’ depression among primary and secondary schools in China. The specific mediating paths and corresponding effect values are presented in Table 3.

**Table 3:** Analysis of the mediating effects in present study

	Effects	Boot SE	Boot ULCI	Boot LLCI	relative effect
Total effect	﹣0.375	0.017	﹣0.4077	﹣0.3431	/
Direct effect	﹣0.17	0.015	﹣0.2002	﹣0.1409	/
Indirect effect	﹣0.205	0.012	﹣0.2283	﹣0.1823	54.67%
Path 1	﹣0.052	0.006	﹣0.0645	﹣0.0397	13.87%
Path 2	﹣0.105	0.009	﹣0.124	﹣0.0871	28%
Path 3	﹣0.048	0.004	﹣0.0564	﹣0.0403	12.8%

Path 1 indicates family support → subjective time pressure → depression; Path 2 indicates family support → work-family conflict → depression; Path 3 indicates family support → subjective time pressure → work-family conflict → depression

## Discussion

A plethora of studies have previously investigated the mechanisms affecting teacher mental health, with a predominant focus on individual and organizational factors. However, the present study delves into the role of family factors in teacher depression and its underlying mechanisms. This center contributes to a more comprehensive understanding of the issue. A comprehensive understanding of the influence of family support on mental health can facilitate the identification of protective factors within the family environment. This can enable the provision of timely psychological interventions for educators accordingly. Moreover, the findings provide substantiation for the COR theory, which posits that familial resources can effectively serve as a buffer against work- and life-related stress, thereby promoting enhanced subjective well-being and mental health.

### The relation between family support and teachers’ depression

Firstly, the results indicated that family support was significantly and negatively associated with teachers’ depression, thereby examining Hypothesis 1. The findings of this study further substantiate the core tenet of social support theory, which posits that when teachers receive enhanced family support in daily life, whether material or emotional, they experience more positive emotional states [5, 41]. A fundamental aspect of depression is the presence of diminished self-worth (Chaurasia et al., 2020). According to Self-Determination Theory, when individuals receive sufficient support in their lives, their basic psychological needs (e.g., relatedness) can be more effectively satisfied [7]. This leads to a greater sense of personal value, thereby promoting psychological well-being. Therefore, support from the family, particularly from spousal support [13], is especially important. Naturally, support from other family members, such as parents, is also crucial. This is particularly true for families with children, where the assistance and support of grandparents are irreplaceable.

### The mediating role of subjective time pressure

Secondly, the findings further demonstrate that subjective time pressure mediates the relationship between family support and depression among educators in primary and secondary school. These findings contribute to the expanding corpus of empirical research on COR theory. Time is a precious and finite resource. According to the COR theory, individuals endeavor to acquire and maintain resources throughout their lives. However, when they are unable to obtain sufficient resources, stress arises, then further depletes an individual's resources and adversely affects their physical and mental health [18]. A review of the extant literature suggests that educators in China typically engage in work hours that extend beyond the legal threshold established at the national level [11, 34]. This finding suggests that when confronted with an objective scarcity of time, teachers who receive limited family support may experience heightened subjective time pressure, leading to greater resource depletion. In order to safeguard their remaining resources, individuals may adopt a defensive posture, which can lead to consequences such as job burnout and depression [22]. Consequently, those with fewer resources tend to encounter more protracted career progression within organizations, a phenomenon that is particularly deleterious to teachers' personal growth and professional advancement.

### The mediating role of work-family conflict

Moreover, the present study found that family support is indirectly associated with teachers' depression via work-family conflict, which is consistent with extant research [17, 35]. The JD-R posits that family support constitutes a critical external resource for individuals in the workplace [10]. Specifically, sufficient family support in teachers' work can effectively enhance their capacity to cope with work-related demands and stress, mitigate work-family conflict, and thereby improve mental health. On the other hand, the work-family enrichment theory posits that when individuals encounter reduced levels of work-family conflict and depression, the knowledge and skills acquired in the workplace may serve to fortify their family roles, thereby engendering a virtuous cycle between work and family (Greenhaus & Powell, 2006). That said, insufficient or absent family support forces teachers to balance family responsibilities and teaching duties simultaneously, which may exacerbate work-family conflict and depression [33]. Thus the strategic allocation of family resources has the potential to foster both the mental well-being of educators and the enhancement of familial harmony.

### The mediating role of work-family conflict

The findings indicate that teachers' subjective time pressure intensifies work-family conflict, thereby exacerbating depressive symptoms. This finding lends empirical support to the COR theory [18]. When individuals perceive heightened subjective time pressure, they mobilize additional resources (e.g., time, cognitive) to cope. However, this can impair cognitive function and diminish their ability to effectively manage work-family conflict [39]. Role theory posits that as individuals assume multiple social roles and the associated expectations that accompany them, time pressure can intensify conflicts between the domains of work and family. This, in turn, can have a negative impact on an individual's physical and mental health [23]. Consequently, a reduction in teachers' working hours coupled with a moderation of their role expectations may prove an effective means of alleviating work-family conflict.

### Limitations and future directions

This study investigates the antecedents and mechanisms of depression among primary and secondary school teachers in southwest China from a familial perspective. The study offers theoretical insights and practical implications for safeguarding and enhancing educators' mental health in the region. However, it is imperative to acknowledge the limitations inherent in this approach.

Firstly, the study employed a cross-sectional design, capturing the measured variables, such as family support and depression levels, at a single point in time. While this approach identifies associations among constructs, it precludes causal inferences. For instance, although a negative correlation was observed between family support and depression, it remains unclear whether stronger support leads to reduced depression, or whether lower depression facilitates the perception of greater support. Furthermore, unaccounted confounding variables (e.g., personality traits, coping strategies) may underlie the observed relationships. Future studies should adopt longitudinal or experimental designs to trace temporal dynamics, establish causality, and examine how changes in family support influence trajectories of teacher mental health over time.

Secondly, the sample was restricted to teachers within Sichuan Province, which may limit the generalizability of the findings. Regional characteristics, including local education policies, economic conditions, and cultural norms regarding family roles, have the potential to influence both the perception of support and mental health outcomes in ways that may not be representative of all Chinese teachers. Therefore, it is imperative to exercise caution when extrapolating the findings to other regions, particularly those characterized by distinct socioeconomic and cultural profiles, such as coastal metropolitan areas or less developed regions. In order to enhance external validity, future research should utilize stratified sampling across diverse geographic and socioeconomic contexts, including both urban and rural settings from multiple provinces.

Thirdly, this study operationalized both family support and depression as single-dimensional constructs. To this end, scales were employed that aggregated support from diverse family members and various depressive symptoms into composite scores. However, both concepts are inherently multidimensional. The concept of family support encompasses a variety of forms of assistance, including emotional companionship and domestic sharing from spouses, childcare and financial aid from parents, and instrumental support (e.g., extensive grandparental involvement in childcare, a common phenomenon in many Chinese households). The availability, frequency, and psychological impact of these forms of assistance may vary significantly. Depression is characterized by three interconnected dimensions: cognitive, somatic, and affective. The application of global measures has the effect of obscuring potential differential relationships between specific types of support and particular symptom clusters. Accordingly, subsequent studies should aim to disaggregate these constructs, distinguishing between sources of family support and dimensions of depression, to examine their nuanced associations. This sophisticated strategy can facilitate the identification of the forms of support that are most effective in mitigating specific aspects of depression. Consequently, it can enable the development of more targeted and effective mental health interventions.

## Data Availability

The datasets analyzed in the present study can be obtained from the corresponding author upon reasonable request.
